# How the brain controls decision making in a multisensory world

**DOI:** 10.1098/rstb.2022.0332

**Published:** 2023-09-25

**Authors:** Christopher R. Fetsch, Uta Noppeney

**Affiliations:** ^1^ Solomon H. Snyder Department of Neuroscience, Zanvyl Krieger Mind/Brain Institute, Johns Hopkins University, Baltimore, MD 21218, USA; ^2^ Donders Institute for Brain, Cognition and Behaviour, Radboud University, 6525 EN Nijmegen, Netherlands

**Keywords:** multisensory integration, decision making, neuroscience, perception, metacognition, causal inference‌

## Abstract

Sensory systems evolved to provide the organism with information about the environment to guide adaptive behaviour. Neuroscientists and psychologists have traditionally considered each sense independently, a legacy of Aristotle and a natural consequence of their distinct physical and anatomical bases. However, from the point of view of the organism, perception and sensorimotor behaviour are fundamentally multi-modal; after all, each modality provides complementary information about the same world. Classic studies revealed much about where and how sensory signals are combined to improve performance, but these tended to treat multisensory integration as a static, passive, bottom-up process. It has become increasingly clear how this approach falls short, ignoring the interplay between perception and action, the temporal dynamics of the decision process and the many ways by which the brain can exert top-down control of integration. The goal of this issue is to highlight recent advances on these higher order aspects of multisensory processing, which together constitute a mainstay of our understanding of complex, natural behaviour and its neural basis.

This article is part of the theme issue ‘Decision and control processes in multisensory perception’.

## 1. Introduction

Precise and accurate perception in a dynamic multisensory environment poses fundamental computational challenges for the brain. Faced with a multitude of incoming signals, the brain needs to solve the causal inference or ‘binding’ problem—inferring which signals come from a common source and thus should be integrated. At the same time, it must also estimate the reliability of each signal to determine how much influence it should have on the perceptual judgement. Effective decision making thus requires the brain to grapple with the uncertainty associated with various sensory signals as well as their causal relationship. Recent work suggests that the brain can monitor the degree of uncertainty associated with various sources of information and actively control whether, when and how this information is integrated. Other top-down processes are critical for regulating sensory integration according to learned categories, goals/expectations and motor intentions. Each of these factors contributes to a decision process that unfolds in time with complex dynamics, flexible strategies and metacognitive assessments. This theme issue brings together contributions from leading experts who seek to understand the computational and neural underpinnings of multisensory cognition, paving the way for a mechanistic understanding of perception and decision making in natural environments.

## 2. Overview of the theme issue

The contributions include both review articles and primary research, from groups that span a diverse set of techniques and conceptual/theoretical frameworks. They range from single-unit and neural population recordings in animals (monkeys, rats and mice) to functional magnetic resonance imaging and electroencephalography (EEG) in humans, often accompanied by psychophysics and computational modelling. We have organized the contributions into three broad subthemes.

### (a) Temporal dynamics and decision processes in multisensory perception

Sensory information is imperfect, owing to limitations in our sensory apparatus, and to noise in both the physical events being transduced and the neural representation thereof. For this reason, perceptual judgements are productively studied within a framework of decision making under uncertainty. Indeed, statistical decision theories have featured prominently in psychophysics for nearly a century, a tradition that carries forward into many corners of multisensory research.

A common approach is to frame the output of the perceptual system as a continuous estimate of the relevant environmental variable, leading to frameworks such as maximum-likelihood estimation. Ultimately, however, a percept must guide discrete behavioural choices, invoking the concept of a decision as the application of a rule or policy to the sensory evidence. Moreover, the evidence usually arrives not as a single snapshot but a temporal sequence or stream of information, and therefore we must decide not only *what* but also *when* to commit to a decision. This perspective has a track record of success in the study of perceptual decisions based on individual modalities [1,2], but only recently has it penetrated the multisensory field.

Zeng *et al.* [3] review basic elements of ‘multisensory decision making’ as a novel application of perceptual decision frameworks (e.g. signal detection theory and evidence accumulation/drift-diffusion) in a multisensory context. They describe a set of classic studies on visual-vestibular integration for self-motion (heading) perception, and more recent attempts to understand how signals are integrated both across modalities and over time—a computation made more difficult by the diverse dynamics of the constituent signals. At the core of this line of work is an intriguing mystery: several mid-level sensory cortices appear to combine visual and vestibular signals, but the ‘read-out’ (both behaviour and neural activity in downstream decision-related areas) suggests that the decision process may bypass these intermediate representations and instead sample information from earlier, unisensory regions.

Jerjian *et al.* [4] continue the focus on decisions about self-motion, charting the evolution of the field from two-alternative forced choice psychophysics to more complex, naturalistic tasks. They also describe preliminary findings connecting the phenomenon of reliability-based cue weighting with measures of response time and decision confidence, arguing that the latter are important ingredients for constructing sequences of decisions such as during navigation. Confidence itself is considered a form of metacognition (thinking about thinking) [5], the study of which (see below) is rapidly advancing in both humans and animal models. Interestingly, the new results [4] support the idea that confidence and cue combination may draw upon a common estimate of sensory uncertainty, despite the former being considered metacognitive and the latter a more automatic perceptual process (see also [6]).

Zaidel & Salomon [7] draw much needed attention to the distinction between the self and the environment, offering a nuanced take on interoception and active sensing in the context of multisensory decisions. Along the way they expose a profound limitation in the conventional way of thinking about (and testing) multisensory integration: in short, there are no true ‘unisensory’ conditions, and thus experiments and models that compare unisensory and multisensory performance can be subject to fundamental misconceptions.

Choi *et al.* [8] offer a comprehensive review of the anatomy and physiology of multisensory integration in mammals, illustrating how multi-modal processing is utterly widespread throughout both the cerebral cortex and subcortically, and how these complex brain networks may be disrupted in neurological and psychiatric disease. The flexibility of routing and integration in these networks should feature prominently in our best theories of perceptual decision making and could be the key to unlocking new therapeutic strategies as well.

### (b) Top-down influences on multisensory processing

Psychology has long grappled with the nature and extent of ‘top-down’ influences on perception [9–11], and neuroanatomy alone tells us that sensory pathways receive extensive feedback and recurrence even at early stages [12–14]. A new surge of interest in this topic centres on exactly how the synthesis of multisensory information is governed by these higher order signals.

Pennartz *et al.* [15] zero in on the visual cortex in rodent models, marshalling an extensive body of evidence in support of the idea that this region is not, in fact, purely visual. Rather, it incorporates signals from motivational, reward and motor systems, in addition to other sensory modalities. They conclude that visual perception emerges from interactions between these systems, encompassing a large network of brain regions, and that these interactions can be understood within the framework of predictive processing.

Stange *et al.* [16] investigate the influence of crossmodal predictions on neural processing in visual cortices by training participants to associate different sounds with spatially specific visual signals. Using EEG, they show that when presented alone these sounds elicit spatially specific responses only at later processing stages but do not elicit the early visual evoked potentials such as the C1 response. These results suggest that top-down crossmodal predictions and bottom-up visual stimulation influence neural processes in visual cortices through partly distinct neural circuitries.

In one of the most extreme examples of top-down influence on sensory systems, Groh and colleagues [17] discovered that information about eye movements travels all the way to the auditory periphery: saccades are accompanied by systematic, transient, oscillatory deflections of the eardrum. Whether driven by cochlear efference (outer hair cell motility) or by innervation of the middle ear muscles, these oscillations are speculated to facilitate coordination between visual and auditory systems for spatial localization of stimuli. Here, Lovich *et al.* [18] extend this fascinating story, elucidating quantitative relationships between eardrum oscillations and saccade metrics in both humans and monkeys. They suggest that similarities and differences in these relationships across species will help guide generation of hypotheses about the functional role of this surprising phenomenon.

Newell *et al.* [19] explore the interplay between multisensory perception and category formation across the lifespan. They argue that category formation is informed by redundant (e.g. shape from vision and touch) and complementary information (e.g. a wolf's colour and its vocalization) that may be integrated at early and late stages during multisensory perception. Conversely, conceptual information extracted from the sensory inputs and stored in semantic memory can in turn influence multisensory processing during perception.

Vocal communication is a canonical example of a behaviour that depends heavily on visual-auditory integration—just imagine locating your friend at a crowded party, or tracking the singer's facial movements to better apprehend the lyrics at a rock concert. Romanski & Sharma [20] review the neural mechanisms of multisensory decisions concerning conspecific vocalizations, focusing on the monkey ventrolateral prefrontal cortex (a likely homologue of human inferior frontal gyrus, which includes Broca's area). Beyond merely reflecting the auditory content, signals in the ventrolateral prefrontal cortex integrate contextual information to construct a representation of the identity and facial expression of the speaker, forming the basis for many essential features of our behaviour as social primates.

### (c) Uncertainty, causal inference and metacognition

Perceptual inference and decision making requires the brain to deal with multiple sources of uncertainty [21,22]. Numerous studies have shown that the brain integrates noisy sensory information weighted by its precision (i.e. inverse of uncertainty) in accordance with normative Bayesian principles [23,24]. This makes multisensory integration a prime example of Bayes-optimal behaviour. It contrasts with the frequent deviations from optimality observed in higher order cognitive decisions.

Aston *et al.* [25] propose that these differences may partially arise from the distinct types of uncertainty involved in perceptual and non-perceptual decisions. While perceptual decisions primarily face internal uncertainty arising along sensory organs and pathways, non-perceptual decisions grapple with external uncertainty such as those emerging from a dynamically changing world. To test this hypothesis, the researchers conducted an audiovisual spatial decision experiment, manipulating both internal and external uncertainty. The results confirm that humans account for internal uncertainty more effectively than external uncertainty during audiovisual integration.

Importantly, the brain should integrate sensory signals only when they originate from common sources, but segregate them otherwise. This computational challenge has been coined the causal inference problem [26]. Behavioural, computational and neuroimaging research has shown that the brain arbitrates between sensory integration and segregation in line with Bayesian causal inference principles [21,27–29]. Most notably, observers integrate audiovisual signals when they are close in space and segregate them at greater spatial disparities [26].

Marly *et al.* [30] explore whether this arbitration between sensory integration and segregation involves conflict monitoring and cognitive control. In support of this conjecture, the researchers observed longer response times and greater theta oscillatory power at intermediate audiovisual spatial disparities when observers are least certain about whether signals come from common or independent sources.

Badde *et al.* [31] examined causal inference in more complex visuo-haptic settings that incorporate two object features, slant and roughness. The degree to which visuo-haptic roughness and slant features were integrated optimally varied across participants in an uncorrelated manner, suggesting that causal inference is performed at the feature- rather than object-level.

Noel *et al.* [32] extend causal inference further to more naturalistic settings in which perception is a continuous perception-action loop rather than a simple stimulus-response mapping. They derive a normative model for how agents should navigate towards a moving target, which requires a causal inference step to disambiguate retinal motion due to self- versus object motion. They find that navigational and eye-movement trajectories are consistent with a dynamic causal inference process whose underlying computations unfold in time, furnishing a platform for studying the neural basis of causal inference in naturalistic behaviour.

Ample evidence from simple unisensory perception has shown that observers not only account for uncertainty during perceptual decision making, but also metacognitively monitor their own uncertainty [5]. Multisensory integration and causal inference raise new challenges and questions for metacognition [33]. For instance, are observers metacognitively aware of the signals' causal structure? Do they feel less confident about perceptual outcomes when sensory signals provide conflicting evidence? Using the sound-induced flash illusion, Maynes *et al.* [34] show that observers are more confident about their veridical than their illusory two-flash percepts. These results suggest that causal inference influences perceptual uncertainty, leading to lower confidence when auditory and visual signals conflict.

Lastly, Meijer & Noppeney [35] explore the relationship between causal and perceptual inference and metacognitive confidence. Using the McGurk illusion in a dual-task paradigm that combines perceptual and causal decisions along with associated confidence ratings, they show that the outcomes and confidence levels associated with perceptual and causal inference are closely related over trials, as predicted by Bayesian causal inference. Notably, veridical and illusory ‘Ga’ percepts were associated with comparable perceptual and causal confidence. This suggests that multisensory integration of conflicting and congruent audiovisual signals can lead to perceptual and causal metamers that are metacognitively indistinguishable.

## 3. Conclusion

Sensory integration is fundamental to life. Adaptive behaviour requires organisms to combine inputs furnished by various sense organs to make decisions and generate motor outputs. Despite the increasing pace of multisensory research in recent decades, we have only just begun to understand how the brain can monitor and actively control the integration of diverse streams of information.

This theme issue has brought together research and reviews highlighting new important directions. The first group of contributions highlights the dividends of framing multisensory integration as a decision process, including an appreciation of temporal dynamics, mechanisms for flexible routing of information and guidance of hierarchical or sequential decisions. Looking ahead, the move towards more naturalistic paradigms will shift the focus towards active sensing across multiple modalities. The second group of studies emphasizes complex interactions between bottom-up multisensory processing and top-down mechanisms related to attention, cognitive control and memory. The final set of contributions focuses on how the brain flexibly integrates and segregates sensory signals in the face of uncertainty about the environment's causal structure, and how perceptual and causal uncertainty constrain our ability to reason about our own mental processes (metacognition).

Although far from comprehensive, this snapshot of an evolving field derives credibility from its breadth of model systems. The hope is that collectively we might bridge across phylogeny to identify core computational principles of perception and cognition, and to uncover both conserved and unique biological underpinnings. A multisensory perspective invites a search for mechanisms that translate the ‘language’ of each individual modality into the language of motor control, or of abstract thought. It also requires us to ask how brain circuits can actively control the flow of this information on a rapid time scale, as the sensory environment does not wait around for slow synaptic plasticity, nor does it furnish millions of trials like those used to train artificial neural networks.

Three decades ago, when Stein and Meredith published their classic *The merging of the senses* [36], the principles of multisensory integration seemed relatively tractable in terms of bottom-up pooling operations whose neural basis lay ripe for the picking. Similarly and not long after, the psychophysics of optimal linear cue combination [23,37–39] emerged as the poster child of a revitalized Helmholtzian theory of perception, and a pillar of the ambitious ‘Bayesian Brain’ hypothesis [40]. These early forays remain indispensable, but the scope and complexity of our questions has expanded. This creates challenges but also exciting opportunities for a new type of merger: one that synthesizes (or obviates?) concepts of bottom-up and top-down processing to reveal how such radically diverse streams of information cohere into unitary experience and purposive action.

## Data Availability

This article has no additional data.
